# Black Robes and White Coats: Daubert Standard and Medical and Legal Considerations for Medical Expert Witnesses

**DOI:** 10.7759/cureus.69346

**Published:** 2024-09-13

**Authors:** Joseph Pergolizzi, Jo Ann K LeQuang

**Affiliations:** 1 Pain Medicine, NEMA Research, Inc., Naples, USA; 2 Scientific Communications, NEMA Research, Inc., Naples, USA

**Keywords:** daubert standard, frye standard, lawsuits, legal medical case, medical expert witness

## Abstract

Expert testimony can play a pivotal role in a legal case involving a medical issue, but it is crucial that this testimony be scientifically sound. In the United States, the Frye Standard demanded that such expert medical testimony be “generally accepted,” but it has been superseded by the more demanding Daubert Standard. Under Daubert, judges became “gatekeepers” as to what scientific material was admissible in court and what might be dismissed as junk science. While a vast improvement over Frye, the Daubert Standard still faces its own problems. Judges usually lack scientific and medical expertise and sometimes have to do their own medical research to be able to evaluate the experts and their testimony. Expert witnesses can be subjected to an adversarial Daubert challenge, even during trial, to establish their credentials and the evidence they are reporting. About half of all judges have had no formal education in handling scientific evidence, but 91% felt comfortable in their gatekeeper role. Considerations for expert witnesses include training or coaching, familiarity with the entire case and courtroom procedures, and an ability to not just know the evidence but to be able to communicate it effectively to a lay audience (jury). Some countries have pioneered a concurrent testimony approach in complex cases, whereby experts argue the evidence of the case under oath before a judge in advance of the trial to determine where there are points of agreement and where the main areas of contention reside. This process, colorfully called “hot tubbing,” is aimed at a more conciliatory approach to reaching compromises. Nevertheless, bias still can result even in hot-tubbing cases, and more research is needed to better understand the nature of jury bias and how it can affect jury decisions in complex medical cases.

## Introduction and background

When the Daubert Standard replaced the Frye Standard for expert testimony in federal United States courts in 1993, it transitioned the role of the federal judge from one who accepted testimony from recognized experts to become a “gatekeeper” as to what evidence was credible [1]. Under the Frye Standard, judges were permitted to admit testimony from recognized and credible experts without having to weigh in on the actual substance of the testimony; it required judges to vet credentials rather than evidence. The Daubert Standard vastly expanded this ruling to the effect that judges are expected to be able to consider the validity of specific forms of evidence.

As the intersection between medicine and jurisprudence widens and deepens in complexity, physicians and other medical scientists increasingly find their way into the legal terrain, including the courtroom. Few physicians have ever been trained to give expert testimony in federal court. Indeed, there are few training opportunities or guidelines for this important work [1]. The same dearth of specialized training applies to judges, who may be called upon to assess the validity and reliability of complex medical evidence, in which they have little training or education.

Expert witnesses and their testimony in medical cases must meet the Daubert Standard to be allowed to offer testimony. Opposing counsel can disqualify an expert witness, even in the middle of a case, with a successful Daubert challenge. This type of challenge does not apply to fact witnesses in the trial, but expert witnesses enjoy both risks and privileges that fact witnesses do not. In most cases, expert witnesses have broad latitude to offer opinion and speculation and may be permitted to use otherwise inadmissible evidence in drawing their conclusions. Fact witnesses are permitted to testify only as to what they specifically saw or heard. In addition, expert witnesses enjoy a high status with most jurors, who assume that their expert status has been properly confirmed by the court [2].

Physicians, scientists, or other healthcare professionals called to a deposition or to provide expert testimony in a legal case may be unaware of the Daubert Standard, how it might be applied, and why it is in use. The aim of this narrative review was to explore the Daubert Standard in light of legal proceedings that may require healthcare professionals to testify.

Methods

On June 10, 2024, PubMed was searched for “Daubert” and “Daubert Standard,” which yielded 211 results. The bibliographies of those articles were searched, as were websites offering articles on expert testimony. This narrative review focuses on offering healthcare professionals specific considerations on whether they should be called to serve as expert medical witnesses.

## Review

Expert testimony often plays a pivotal role in legal cases about medical issues, but such testimony must be based on evidence rather than opinion or even clinical experience. Most medical testimony relies on data from published clinical trials, although other forms of scientific evidence may be accepted as well. 

The earlier Frye Standard emphasizes that evidence, in order to be legally admissible, had to be generally accepted. The subsequent Daubert Standard, which is utilized in federal courts and most state courts in the United States, reiterates and expands on this fundamental concept [3]. A potential shortfall of the Frye Standard is that certain widely accepted medical concepts may be scientifically unproven; an example is the six-foot distancing rule during the COVID pandemic. By the same token, there are medical concepts that have not been fully elucidated, such as the cause of migraines. Thus, the Daubert Standard imposed new premises for the court, summarized in Table 1 [4]. Under the Frye Standard, it was only the last point-widespread acceptance that determined the admissibility of expert testimony [5].

**Table 1:** The main premises for judges to consider in the Daubert Standard. Note that the judge has discretion in terms of how much weight is given to each premise, but a judge may not use one criterion in isolation to allow an expert witness to testify.

Premise	Comment
Can the theory or technique be tested? Has the theory been tested?	Falsification testing, that is, tests that confirm the validity of key assumption(s)
Is the evidence published and has it undergone peer review?	While this is part of the Daubert Standard, the judge has discretion in terms of whether or not this is considered and how much weight it is given
What is the error rate?	Error rate is determined by the sample size and study design, which can be complex and often require an understanding of statistics
Were there standard controls in place?	These controls should be clear and maintained over the course of the study
Has this theory or technique gained widespread acceptance in the relevant medical or scientific community?	No definitions are offered for these terms: “widespread,” “acceptance,” and “relevant community”

The transition from the Frye to Daubert Standard established the role of the judge as “gatekeeper” for expert testimony, and it also set up the Daubert challenge, in which opposing counsel may try to disqualify an expert witness.

Two cases subsequent to the Daubert ruling in 1993 have been formative in helping shape the application of the Daubert Standard today. One is General Electric Co. v. Joiner (Joiner), and the other is Kumho Tire vs. Carmichael (Kumho) [6]. In abbreviated terms, the Supreme Court in Joiner ruled that the conclusions drawn by experts had to be adequately supported by the studies on which they were based and that those studies had to be similar to the case. In other words, the Daubert Standard was not met if an expert witness used a study unrelated to the present case and/or if the opinions expressed by the expert witness were not solidly supported by the study. In Kumho, the Supreme Court allowed that judges could take into account the professional experience of their expert witnesses along with the evidence they presented.

Other court decisions have modified the Daubert interpretation. In United States v. Mooney, it was ruled that once the judge had determined that an expert witness was reliable, used sound scientific principles and methodology, and validated reasoning, then that expert witness was free to offer inferences, opinions, and draw conclusions, something not afforded to fact witnesses [6].

Expert witnesses

Rule 702 of the Federal Rules of Evidence was amended in 2000, requiring that expert testimony be based on specific data or facts; that the testimony is the result of using reliable methods and principles; and that the expert witness applied those principles and methods reliably to the facts in the case. Rule 702 thus excludes “expert opinion” and differentiates it from “expert testimony,” although expert witnesses are allowed some leeway in offering their opinions from the witness stand.

It should be noted that clinical experience, although not an obstacle to expert witness testimony, was not sufficient in and of itself to confer expert status on a witness. In fact, in court proceedings, clinicians who have direct clinical experience and have made relevant clinical observations may be excluded as expert witnesses because their level of expertise may be insufficient for judicial standards per Daubert [7]. In some medical cases, healthcare professionals may be called in as fact witnesses rather than expert witnesses because they testify as to what they saw, heard, or otherwise observed, such as a patient’s condition, the medical intervention performed, or how long a procedure took. Thus, a factual witness is held to a lower standard than an expert witness.

Expert witnesses may be called by the defense or prosecution, and in some cases, the court itself will call a specific expert.

The Daubert challenge

In a legal proceeding, the identity of expert witnesses is typically announced prior to the case, and opposing counsel has the right to issue a Daubert challenge to possibly preclude the testimony or exclude the expert based on the Daubert premises shown in Table 1. While a Daubert challenge is most often brought before the beginning of the trial, it may also be brought as a separate motion, part of a summary judgment, a motion in limine, a post-trial motion, or as part of an objection to testimony by the expert. This means that an expert witness may be scrutinized using the Daubert challenge at any point during or even immediately after the trial.

The Daubert Standard requires that the testimony of the expert witness be based on clinical studies or other forms of research that were set up based on a testable scientific hypothesis [8]. The expert need not have conducted that actual research but must provide testimony congruent with such studies. To prepare for a Daubert challenge, the expert witness should be thoroughly familiar with the relevant clinical data, including the study hypothesis, study design, sample size, how the sample was derived, sample demographics, randomization schemes, and results. In depositions, witnesses are typically counseled to be succinct, but in a Daubert challenge, it may be helpful for the expert witness to offer more detail to better explain the type of study, how it was conducted, and why that particular study design was chosen. The goal of a Daubert challenge is to help the judge understand the nature of the testimony, so more information may be better than less.

The Daubert Standard also seeks publication of the studies used in peer-reviewed medical journals. The peer-reviewed publication criterion appears to be the easiest test to apply, but results can be deceptive [8]. The expert witness need not be the author of the peer-reviewed articles used in the testimony, although that is often the case. Further, the expert witness need not be a peer-reviewed author at all. However, the expert witness must be thoroughly familiar with all studies on which testimony is to be based.

While the Daubert Standard does not specify how much weight the judge is to give any of its premises, it may be that there is too much emphasis placed on peer review. Peer review is a black box binary procedure where authors either pass or fail; there is no transparency with respect to the issues or discussions that arose in the process. An article may fail peer review on a technicality or by an overly harsh standard. (There are no guidelines for peer reviews other than those offered by a specific publication, and many publications do not even offer that.) Just as bias can enter a legal proceeding, bias can infect the peer review process. For example, a study using artificial intelligence (AI) found that when the first author of a paper was detected as female, the peer review was harsher than if the first author was male [9]. Bias may also appear in favor or against certain medical procedures, since many medical topics can be controversial, such as sexual transitions of minors, abortion, vaccination, obesity ("body positivity"), psychiatric drugs, and medically assisted suicide. In fact, there may be few medical topics that are universally approved.

More concerning than bias is that peer-reviewed publications are being scrutinized since the number of retractions in scientific journals has increased 10-fold over the past decade. These have become so numerous that a website called RetractionWatch.com monitors these events [8]. This surge in retractions may be attributed to any number of factors: the proliferation of new, smaller journals; the surge in predatory publishing; the rise of AI, particularly in generating fake images; and the lack of universal standards in publishing, which is becoming increasingly international. Journals do not always report retractions, much less publicize them. Furthermore, retraction is a broad term and includes articles removed for reasons that have nothing to do with fraud or malfeasance. Some articles are retracted because of problems in stating authorship, conflicts of interest, lack of institutional review board approval, or “honest” errors in data [8]. Note that retracted articles do not have to disclose why they were pulled, and a journal does not need to announce or publicize a retraction. While the status of given medical publications can be established in real time, the increased number of retractions points to a broader issue: do articles need an extra layer of scrutiny before being considered, even if they are published in an established peer-reviewed journal?

The error rate can be a puzzling criterion for non-researchers. While the Daubert Challenge can rightly explore the error rate of the various clinical trials used to support the testimony of an expert witness, in a survey among 400 state court judges, only 4% of the respondents showed a correct understanding of the error rate [10]. In basic terms-which may be useful in a Daubert challenge-the error rate is the difference between the theoretical test results (what the researchers would expect in a perfect world) and the results the study actually obtained [8]. While error rates are simple to understand and highly valuable to consider in medical research, they can be problematic to assess. They are influenced by clinical trial design; they require a definition as to what constitutes an error, and they demand a good understanding of statistical probability and standard deviations. Finding an acceptable error rate may require comparison to other studies and the ecological framework of a given study since there are no hard and fast acceptable error rates, such as 10%, appropriate for all studies [11]. Judges may have to consider study power, sample size, and sampling methodology to determine whether the study's error rate was scientifically acceptable [12].

Further, the Daubert Standard may require that the judge assess whether the method of the study on which the testimony is to be based is generally accepted in the relevant scientific community [8]. In other words, the medical literature may contain studies, case reports, or case series based on controversial methods. An example currently under consideration is whether or not a telehealth assessment made for a psychiatric patient would be acceptable under the Daubert Standard since the use of telehealth consultations in psychiatry is not widely accepted, although it is sometimes used [8].

An expert witness does not have to be able to fully satisfy every point of the Daubert Standard, since judges have discretion in terms of how they apply the standard and how much weight (if any) they give specific premises. The Daubert Standard was not intended to be a checklist of rigid criteria but rather a framework that could be applied on a case-by-case basis to evaluate the reliability of specific evidence in a given case [13]. Nevertheless, the Daubert Standard confers a great deal of responsibility on the judge.

Judges as gatekeepers

Judges act as “gatekeepers” in assessing whether or not medical or scientific testimony is sufficiently valid to be introduced into a court proceeding. The Daubert Standard has moved the burden of evidence assessment from the expert to judges, who, despite broad guidelines, may find it difficult to evaluate complex evidence in a medical field in which they lack expertise [14]. The Daubert Standard, although sometimes difficult to apply, represents a step forward because earlier criteria relating to the admissibility of expert testimony were both murky and unevenly applied [14]. While it may be credibly argued that judges today scrutinize expert testimony more intensely and more consistently than in the past [14], judges may not always have sufficient expertise in certain fields to qualify as adequate gatekeepers [15]. If anything, the role of gatekeeper judges has been expanded in recent years, with the ruling that in terms of expert testimony, the role of gatekeeping extends beyond science to include technology and all specialized fields of knowledge [15].

In carrying out the role of gatekeeper, judges may be forced to conduct library research themselves, or they may be forced to rely on a few doctrinal texts [16]. Many rely on what has come to be known as the “bench philosophy of science,” an attempt to make scientific argumentation objective and clear-cut. In medicine, things are often far less clear-cut than the legal system would prefer [17]. Examples of this may be a diagnosis of mental illness or fibromyalgia, both of which are prevalent conditions that may be diagnosed without a physiologic biomarker, imaging study, or laboratory test. Other diagnoses may rely in whole or in part on patient self-reports of symptoms, which are inherently subjective. 

The question arises as to whether it is fair to ask a judge to be a medical or scientific gatekeeper. After all, the scientific expertise and experience of judges is highly varied. In a survey of 400 state judges, 73% had no experience ever dealing with epidemiological evidence, but 65% had some familiarity with DNA evidence [10]. Less than half of judges (48%) felt their formal education had not prepared them to deal with scientific evidence, yet they were apparently comfortable in this role, with 91% of the judges surveyed saying they felt that their role as “gatekeeper” for medical evidence admissibility was appropriate [10].

Considerations for expert witnesses

Those healthcare professionals who wish to serve as expert medical witnesses may find it difficult to get training or preparation for the steps leading to the courthouse. Expert witnesses are typically recruited by attorneys, although sometimes the court itself may engage an expert, and it is usually the attorney or the court that helps the expert witness navigate the process. Expert testimony can be extremely powerful in certain court cases and may be sufficient to influence the jury’s final decisions [1]. In this context, it must be recognized that perhaps the key trait of an expert witness is one not addressed by the Daubert Standard, namely the ability of an expert to communicate medical concepts clearly and effectively to a lay audience. Jurors must be assumed to lack medical or scientific credentials, and the expert must be able to present information in ways that an average person can understand. Thus, the ability to break down complex medical concepts into understandable terms and to explain scientific premises is paramount for an effective expert witness, perhaps more than even academic or scientific credentials.

An expert witness may be challenged, and such scrutiny may be conducted using the adversarial system of the court. The Daubert Standard is, at most, a flexible guideline and a set of recommendations [6]. Challenges to scientific principles, study design, or other factors should not be taken to mean that the witness fails to meet the Daubert Standard, only that the evidence is being scrutinized [6]. For instance, a pain physician asked to testify as an expert in a trial may be asked to explain basic concepts of pain medicine, differences in acute and chronic pain, the main types of pain treatments, and how testimony related to these principles of pain medicine might be helpful in this case. Expert witnesses are also called to provide reasons as to why they should be expert witnesses, namely by reviewing their qualifications, education, training, credentials, knowledge, and clinical or other related experience.

During a Daubert challenge, the expert witness will likely be cross-examined by opposing counsel. In some cases, this adversarial questioning may involve basic questions such as the nature of the medical specialty, how clinical trials are conducted, definitions of key concepts, a history of the specialty, and individual credentials and experience. Generally speaking, the attorneys are given broad latitude in questioning potential expert witnesses in a Daubert challenge [6]. Rarely will an expert witness make it to the witness stand without undergoing at least some sort of pretrial cross-examination, if not a full challenge [6]. Cross-examination is, by definition, adversarial, and the expert medical witness must be prepared for seemingly aggressive questioning. Expert witnesses are routinely compensated, unlike many other witnesses in the case, and opposing counsel may ask an expert witness about payment for testimony. Such questioning should be anticipated, but it may startle the jury to learn that expert witnesses are paid to appear, unlike fact witnesses.

Expert witnesses must recognize that the judge may impose a “non-scientific” burden on jurors, asking them to reach a clear decision supported by robust scientific evidence that goes beyond a reasonable doubt. Such standards are rarely applied in the world of science, where today’s prevailing theory can be refuted by tomorrow’s evidence [1]. Indeed, medical science does not demand the level of conviction to a particular premise that the law does [17], and expert witnesses must help bridge that gap by explaining how science grapples with the truth differently from the legal system. In other words, expert medical witnesses can only testify to our best medical knowledge at this point in time. They may also need to explain how medicine reached these particular conclusions, but expert witnesses may have to concede that science and medicine are an ongoing search for answers. What medicine knows today will not be the same things that medical science knows tomorrow. However, experts are expected to state what is currently known to a reasonable degree of medical certainty.

While expert witnesses have some degree of freedom in providing their explanatory testimony and even being able to offer medical opinions, they are constrained to stay within the realm of their expertise. Venturing outside these boundaries, particularly by offering a legal opinion as to what laws might or might not apply to a given case, risks disqualification [18].

In some complex medical cases, expert witnesses may be brought for both sides, arguing opposing viewpoints. While expert witnesses generally enjoy credibility with the jury, “dueling experts” can be confusing in court cases and may cause the jury to downgrade expert testimony altogether, particularly when contradictory conclusions are introduced [14]. Even with sound scientific evidence, these "expert wars" can derail a case.

In the aftermath of the COVID pandemic, medical experts, medical science experts, and public health experts have lost considerable credibility [19], but it is unclear how and to what extent this might affect expert witness testimony in court. Since expert witnesses must have credibility to be effective [1], specific tactics may be useful to help bolster credibility. For example, it has been found that physicians enjoy greater credibility in communication with lay audiences when the language and terminology they use reflect that of the audience [20]. This is an important point, as juries tend to be more skeptical of experts and highly technical than judges [21].

Credible expert witnesses must understand the entire case rather than just their testimony because cross-examination that exposes confusion or lack of familiarity with case details will damage the credibility of the expert witness and possibly weaken the case [18]. Expert witnesses may benefit from coaching by attorneys as to courtroom etiquette, appropriate dress, and body language, all of which can play an outsized role in enhancing or detracting from credibility [18]. For example, conservative dress, respectful behavior, not interrupting, and avoiding jargon and slang are recommended [18]. A study of jurors’ perceptions of expert witnesses found that the most important elements juries utilized when conferring credibility on an expert witness were education, years of experience, and their demeanor when responding to questions on the stand [22]. In terms of demeanor, credible expert witnesses were expected to be able to easily explain the science with clarity and confidence, to use narrative or story-telling language in their testimony, and when appropriate, to be able to demonstrate their ideas using aids. The use of narrative language was most associated with enhancing believability [22].

Expert witnesses may be called upon to bridge the gap between medicine and forensics. Although footprint evidence has been used in criminal court cases for well over a century, it is only recently that courts have used podiatrists to present such evidence, and this has given rise to the specialty of forensic podiatry [6]. Forensic medicine includes DNA analysis, but it is expanding today into areas such as cybersecurity and the implementation of facial recognition software [23].

Malpractice and disability adjudication cases often rely on expert medical witnesses, but these cases pose unique challenges because there can be a strong emotional component to the case that overwhelms the purely scientific or medical evidence [1]. In malpractice suits, expert witnesses are usually not asked to testify as to whether a physician’s treatment or conduct in the malpractice case might be considered “reasonable,” which is perhaps a better question analyzing various clinical studies [24].

Finally, expert witnesses may be given some latitude to state their ideas, opinions, and predictions in a way that fact witnesses may not. While this liberty is a privilege reserved for certain types of witnesses only, expert witnesses must be careful to retain credibility when stating an opinion and not appear as if they are speculating or trying to influence the jury [18].

Discussion

While a healthcare professional has the freedom to decline to appear in court as an expert witness, the role of the medical expert in civil and criminal litigation remains vitally important. Despite this crucial role, few healthcare professionals are fully prepared to rise to the challenge if called because there is limited training in this field. Expert witnesses are typically paid for their time, and there are some medical experts who serve regularly as expert witnesses as a part or totality of their occupation. Among the many categories of expert witnesses, including financial experts, security experts, intellectual property experts, social media experts, and others, medical expert witnesses are reported to earn the highest hourly rates [25].

Since experts are the only witnesses who are remunerated for testimony or other services to the court (such as providing documents, offering written assessments, being deposed, and testifying in court), this can lead to the perception that experts are “hired guns” who say what they are paid to say. A good rule of thumb is that the expert witness's duty is, first and foremost, to the court, not to the party making payment.

Just as medicine is recognizing the value of the multidisciplinary team, the legal system might benefit in some cases from engaging a multidisciplinary team of medical experts. For instance, in a case involving a pediatric vaccine injury, testimony may be appropriate from one expert about vaccine safety studies, from another expert on the vaccine adverse events reporting database, from another on vaccine development, and from a pediatrician. Such multidisciplinary panels appear good in theory but may be financially or logistically out of reach for many court cases.

This leads to an interesting approach to some arbitration or litigation known by the colorful term “hot tubbing.” Pioneered in Australia, “hot tubbing” or concurrent testimony brings the judge together with experts from both sides in a non-adversarial meeting aimed at getting the key expert evidence out in such a way that points of agreement can be identified, leaving only the points of contention to be fought over in court. “Hot tubbing” observes certain formalities (it is done under oath), but it seeks to find compromise or reasonable solutions. It has been described as a “discussion among experts,” and it is used in all kinds of cases, not just those needing the testimony of medical experts [26]. Despite the conciliatory descriptions of concurrent testimony, it does not appear to reduce the adversarial allegiances typical in court cases [27]. It is doubtful that concurrent testimony will make much headway in the United States, which seems to prefer an outright adversarial procedure. In addition, the best goal of "hot tubbing" is to winnow down all of the points under consideration to those in dispute versus those to which the parties can reach an agreement. In other words, concurrent testimony may save time in particularly complicated cases.

The main goal of the Daubert Standard is to prevent the admission of “junk science” into a court. For that reason, Daubert succeeded Frye, establishing that more than just general acceptance and expertise were needed. Evidence had to be reliable, reproducible, and from studies conducted to sound scientific standards with reasonable error rates. This is particularly important since jurors have shown limited ability to detect erroneous testimony or use scientific principles to draw conclusions [28]. Few jurors have scientific backgrounds. Ideally, an expert witness should help jurors be able to understand the science and cut through their own biases to weigh the evidence.

In some cases, adept expert witnesses can help jurors reach unbiased decisions, but in other cases, expert witnesses may also introduce their own bias [29]. Bias is so ubiquitous it often goes unnoticed, such as jurors who reject all psychiatric medications or jurors who consider all vaccines safe in all cases. Some jurors may reject most conventional medicine, while others may reject all-natural or complementary medicine. Helping a juror to see past the bias is an important goal of the expert witness, but not one that can be easily achieved. A good legal tactic against bias is to impanel heterogeneous juries since bias in individuals can best be countered by diverse jurors from different backgrounds and socioeconomic statuses [29]. Of course, during jury selection, each side obviously tries to select those who would view the case favorably. Another important weapon is a more in-depth study of how bias can affect decision-making, particularly in the high-stakes environment of jury trials. There is a paucity of research on this important topic.

There is a wealth of literature on the Daubert Standard with respect to specific types of cases, and it is a limitation of this article that it focuses primarily on medical testimony and not cases based on psychological testimony, cases about mental illness, or cases about malingering, which present their own unique challenges. We did not address the small but growing number of studies on gender and credibility in expert witnesses. The Daubert Standard is particularly important in toxic tort cases, which was not addressed because it is a deep and very specific field with its nuances. Our goal was to offer consideration for those healthcare professionals who may be involved in a legal case involving a medical question, either as an expert witness or in some other role.

## Conclusions

Law and medicine converge uneasily in court, where judges seek decisions based on objective truths that can be proven, and healthcare professionals can offer insights based on clinical studies and other medical evidence, which, by definition, have a built-in margin for error and are conceived on complex statistical probability projections. Superseding the old Frye Standard, the Daubert Standard set up a framework by which the judge emerged as the “gatekeeper” in determining whether expert witnesses offered valid, sound scientific evidence or might be representing junk science. Although imperfect, the Daubert Standard set up flexible guidelines by which expert witnesses can frame their testimony. Considerations for those serving as expert witnesses in medical cases include meeting requirements, the ability to present complex medical information in a clear way to a lay audience (jury), and an understanding that expert witnesses are granted broad latitude in what testimony they may offer. Nevertheless, there are few formal or academic programs to educate potential expert witnesses who play a crucial role in healthcare litigation.
